# Multivariate analysis of roadway multi-fatality crashes using association rules mining and rules graph structures: A case study in China

**DOI:** 10.1371/journal.pone.0276817

**Published:** 2022-10-27

**Authors:** Chenwei Gu, Jinliang Xu, Chao Gao, Minghao Mu, Guangxun E, Yongji Ma

**Affiliations:** 1 School of Highway, Chang’an University, Xi’an, Shaanxi, China; 2 Innovation Research Institute of Shandong High-Speed Group, Jinan, Shandong, China; 3 Shandong Hi-Speed Group Co., Ltd, Jinan, Shandong, China; Tongji University, CHINA

## Abstract

Roadway multi-fatality crashes have always been a vital issue for traffic safety. This study aims to explore the contributory factors and interdependent characteristics of multi-fatality crashes using a novel framework combining association rules mining and rules graph structures. A case study is conducted using data from 1068 severe fatal crashes in China from 2015 to 2020, and 1452 interesting rules are generated using an association rule mining approach. Several modular rules graph structures are constructed based on graph theory to reflect the interactions and patterns between different variables. The results indicate that multi-fatality crashes are highly associated with improper operations, passenger overload, fewer lanes, mountainous terrain, and run-off-the-road crashes, representing the key variables of factors concerning driver, vehicle, road, environment, and accident, respectively. Furthermore, crashes involving different severity levels, road categories, and terrain are verified to possess unique association rules and independent crash patterns. Moreover, the proportion of severe crashes caused by a combination of human-vehicle-road-environment factors (43%) is much higher than that of normal crashes (3%). This study reveals that the hidden associations between various factors contribute to the overrepresentation and severity of multi-fatality crashes. It also demonstrates that the crash mechanisms involving multi-fatality crashes and their interactions are more complex at the system level than those for normal crashes. The proposed framework can effectively map the intrinsic link between multiple crash factors and potential risks, providing transportation agencies with helpful insights for targeted safety measures and preventive strategies.

## 1. Introduction

Multi-fatality crashes that cause three or more deaths have been a critical problem for traffic safety in China. The fatality rate of roadway crashes in China reached 23.3% in 2018, significantly higher than that of South Korea (1.3%), the United States (1.2%), and Japan (0.8%) [1]. Reducing the casualties of roadway crashes, particularly multi-fatality crashes, has always been a vital issue in the field of roadway safety. Moreover, extensive studies intended to address potential crash factors have focused on direct countermeasures [2]. The proposed safety measures include airbags, electronic stability controllers, energy-absorbing guardrails, and speed enforcement systems [3–5]. Although these advanced technologies can intuitively reduce crash risk at the initial stage, their safety enhancements encounter bottlenecks in long-term use [6]. In fact, the number of road fatalities worldwide has remained unacceptably high in recent years. Furthermore, behavioral adaptations make eliminating key risk factors difficult with general safety measures, which may even cause unintended negative effects under special traffic conditions [7]. Therefore, efforts are required to investigate the underlying mechanisms related to severe crashes, which is essential for improving the practical effectiveness of safety measures.

Since crashes involve drivers, vehicles, roads, and the environment, it is important to not only strengthen the passive protection of roadways and vehicles but also to better explore the underlying variables that contribute to driving risk and crash severity [8]. Several studies have investigated the causal factors of fatal crashes and their impact on crash characteristics. Considering methodology, parametric methods, such as negative binomial, empirical Bayes, and logit-based models, are most commonly used to analyze the associations among variables of fatal crashes [9–11]. By considering crash severity as a nominal or ordinal variable, several principal causes of severe accidents, such as speeding, driver errors, vehicle failure, and weather conditions, have been identified as key characteristics [12]. These studies have attempted to unveil the potential relationships hidden in crash data to extend the road safety theory. However, Weng et al. pointed out that the predetermined assumption of parametric models makes it difficult to identify potential interactions between several factors [13]. Additionally, the correlations among variables may interfere with crash causation and result in incorrect applicability [14].

Given the aforementioned shortcomings, other studies have been devoted to the safety analysis of serious crash casualties using advanced data mining tools. For example, Wang et al. employed a classification and regression technique to identify the important factors affecting driving risk in terms of driver, vehicle, and road environment [15]. Jiang and Ma combined the XGBoost model with a geographic information system to investigate the influence of macro-level factors, such as geospatial, regional economy, and road characteristics, on fatality rates and discussed the relationships among these major factors [16]. Additionally, fault trees, random forests, support vector machines, and Bayesian networks have been widely used for crash prediction models [17–22]. However, according to established literature, the results obtained by such models only rank several independent variables, which hardly reflects the interactions among explanatory variables [14]. This undoubtedly limits the practical application of multivariate crash analyses. In contrast, association rules mining (ARM) has been considered an effective method for discovering potential interactions between human/vehicle features and highway/environment factors, which play a decisive role in the occurrence and severity of fatal crashes. With an understandable rules framework, ARM advantageously identifies fuzzy patterns and heterogeneous effects among several variables in large databases [23]. Moreover, it has been widely used for multivariate analysis of crashes involving rainy weather [24], hazardous material vehicles [25], pedestrian collisions [26], roundabouts [27], and run-off-the-road (ROR) [28].

Although association rules have become a prevailing approach for multivariate analysis of roadway safety, there are still some limitations in applying ARM in practice. The first limitation is the interpretation of rules in ARM. The analysis of interactive associations among variables has gained hardship due to the proliferation of rules generated from existing crash datasets. Most related studies displayed rules with high levels of several indicators while neglecting an in-depth analysis of the overall rules set. However, the minority of rules selected can provide little instinctive and plausible advice to improve road safety [29]. The other limitation of ARM is identifying the critical factors for several crash patterns. It is worth noting that there exist several elevating variables hidden within the rules [28]. Individual elevating variables may not link to the rules satisfying the highest indicators of ARM, but they demonstrate a significant lift on crash patterns when interacting with other variables. These key variables could be elicited from the association rules, which have always been ignored. Graph network theory and analysis is a mathematical method for exploring the interrelations between multiple factors. It has been widely used in behavioral science to analyze social relations, telecommunication science, and transportation [30,31]. Quantitative network analysis is often used to deal with a limited knowledge of the complex relations using graph properties such as topology modularity and centrality measures, which contribute to feature clustering and factor ranking [32]. Moreover, the graph-based visualization presents explicit explanations for complex relationships in association rules. Therefore, this study aims to address the limitations of ARM with the novel rules graph structures (RGSs) based on graph theory.

Considering the aforementioned findings, research that employs rule mining tools to explore hidden associations in severe multi-fatality crashes is limited. To the best of our knowledge, although many researchers have identified the major variables that influence fatal crashes, their interdependencies remain unclear. It seems appropriate to combine association rules and graph theory to describe the relationships among different factors clearly. Furthermore, prevailing countermeasures for normal crashes may not be applicable to reducing serious casualties due to the complex mechanisms of severe accidents [33]. Since the responsibility for any crash involves a range of variables, casualties can be effectively controlled by changing some of them. Therefore, the main objective of this study was to discover the contributory variables and interdependent characteristics of multi-fatality crashes by mining interesting rules. Accordingly, a case study was conducted using 1068 roadway crashes that caused three or more deaths in China from 2015 to 2020. With the utilization of novel rules graph structures, the hidden patterns and interactions among crash factors in rules can be investigated better. This interpretation will present meaningful insights into the selection of preferred countermeasures to reduce roadway casualties.

## 2. Research framework

**Fig 1:**
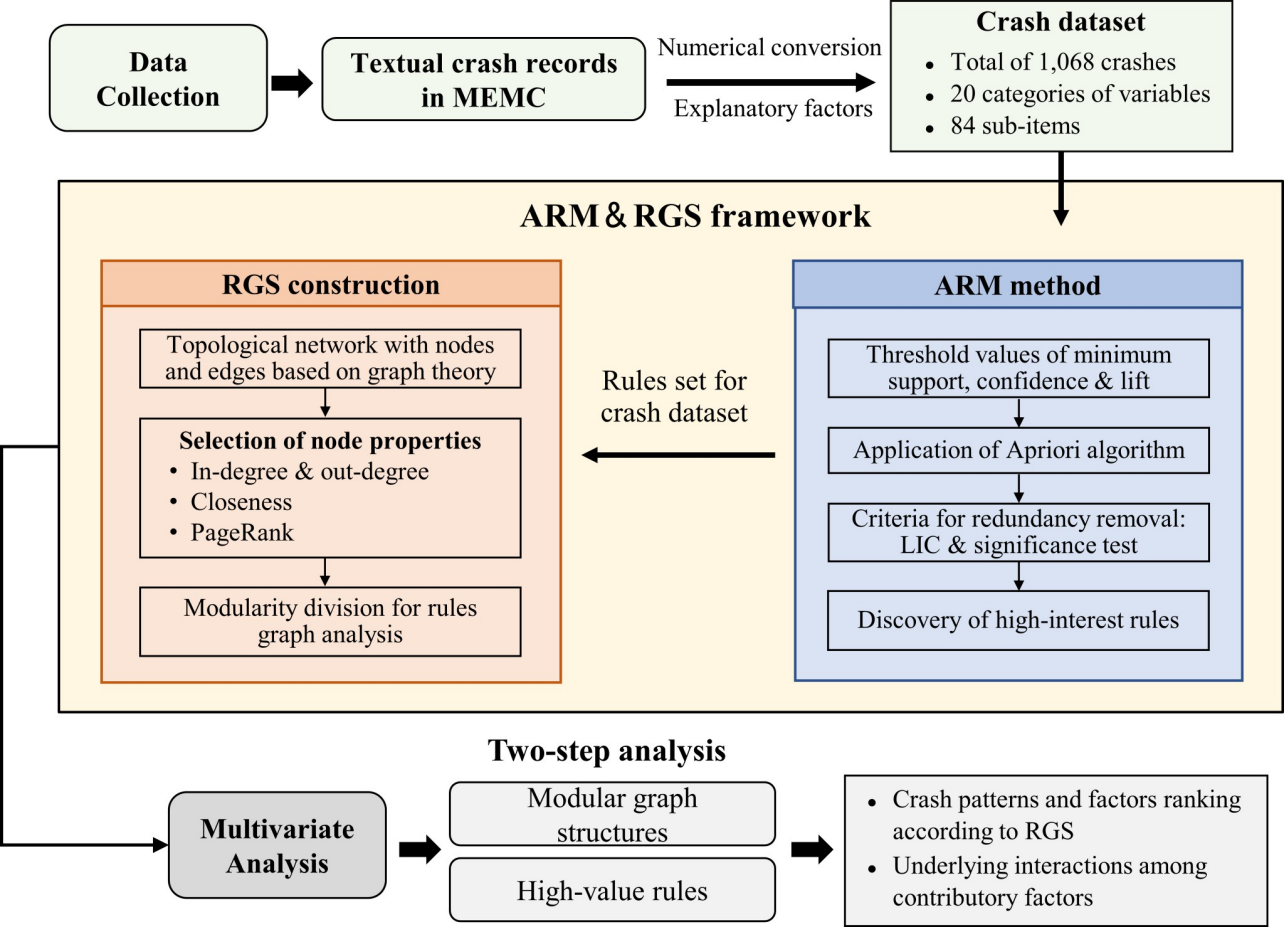
The framework of ARM & RGS method.

This study proposes the ARM and RGS frameworks for multivariate analysis of the contributory factors related to multi-fatality crashes. As shown in Fig 1, the framework consists of four steps: (1) data preprocessing, (2) association rules mining, (3) rules graph construction, and (4) multivariate association analysis. First, the textual records of multi-fatality crashes in China are converted into numerical formats to create a dataset with comprehensive explanatory factors. Second, the Apriori algorithm is applied to discover interesting rules with appropriate threshold values, and several criteria are established to remove redundant rules. Third, the obtained rules are constructed into RGSs based on graph properties and network modularity. Moreover, a two-step analysis is conducted to investigate typical crash factors and patterns using graph structures and high-value rules. With a large number of rules, the interpretation method of rule sets is particularly critical for multivariate analysis. Therefore, the proposed framework attempts to better understand the key crash factors hidden in the rules by constructing a graphical structure. The detailed method is described next.

## 3. Data

In China, for crashes resulting in three or more deaths, a special investigation group of the Ministry of Emergency Management of China (MEMC) at the provincial or municipal level will be deployed to conduct comprehensive investigations on crash-related conditions. An in-depth accident report will be publicly available within 60 days of a crash. These in-depth investigations provide a textual reconstruction of the crash situation, containing detailed information about drivers, vehicles, roadways, and environment factors, which are suitable for the multivariate analysis of accidents.

All crash reports available from MEMC between 2015 and 2020 were collected in this study to determine the factors contributing to multi-fatality crashes and their interactions. Of the total of 1068 crashes used for analysis, 35.3% are significantly serious crashes with more than five fatalities, and 64.7% are severe crashes with 3–5 deaths. These reports include crashes that occurred in 34 provinces and regions, covering all four geographical regions of China. The crash types in the dataset include not only single-vehicle crashes, such as ROR, falling, and rollover, but also multi-vehicle crashes, such as rear-end, angle, and head-on collisions. As in-depth investigations of high quality have been conducted by MEMC professionals and are available to the public, the analysis based on them is reliable.

The following specific patterns are included in the reports for describing crash information:

1. Driver factors, including driver conditions (familiarity, fatigue, and impaired), driving violations, and behavior;
2. Crash information, containing crash location, crash type, fatalities, time period, and number of vehicles involved;
3. Vehicle factors, including the type of vehicle involved, vehicle condition, traveling speed, and deformation of the vehicle;
4. Road conditions, including road category (reflecting the access control and median design of roadways), alignment, road section, number of lanes, and posted speed limit;
5. Environmental conditions, including weather conditions, terrain, traffic conditions, light conditions, and geography.

After careful examination and extraction, all valid textual information from the 1068 crash reports is encoded as a numerical dataset that can then be applied to association rules mining. The dataset includes 18 categories of variables with 84 sub-items. Table 1 lists the factor categories and descriptive statistics for the roadway multi-casualty dataset. All interpreted sub-items are converted into Boolean type for further analysis.

**Table 1:** Descriptive statistics of crash data.

Variable	Count	%	Variable	Count	%
Severity level			Crash location		
A (Casualties: 3–5)	691	64.7	Roadside	335	31.36
B (Casualties > 5)	377	35.3	Roadway	672	62.9
**Time**			Barrier/shoulder	63	5.9
Workday	613	57.4	**Environmental condition**		
Weekend	455	42.6	Rain	233	21.8
0:00–6:00	220	20.6	Snow	54	5.1
7:00–12:00	268	25.1	Wet/slippery surface	303	28.4
13:00–17:00	316	29.6	**Deform**		
18:00–23:00	257	24.1	Disabled	827	77.43
**Terrain**			Functional	203	19.0
Plain	575	53.8	Minor	37	3.5
Mountainous	493	46.2	**Violation**		
**Number of vehicles (Veh num)**			Unlicensed	114	10.7
Veh num = 1	236	22.1	Alcohol	138	12.9
Veh num = 2	583	54.6	Speeding	331	31.0
Veh num = 3	249	23.3	Improper lane usage	352	33.0
**Vehicle type (Veh type)**			Insufficient distance	251	23.5
Light duty	304	28.5	Improper operations	250	23.4
Truck involved	452	42.3	Illegal overtaking	308	28.4
Bus involved	365	34.2	Dangerous driving	194	18.2
**Light condition**			Passenger overload	30	28.1
Daylight	580	54.3	Overspeed > 20%	326	30.5
Dark	228	21.3	Overspeed < 20%	193	18.1
Dark without light	260	24.3	Low speed	19	1.8
**Road segment**			**Limit speed**		
Curve	126	11.8	> 80 km/h	423	39.6
Uphill	44	4.1	[60–80] km/h	375	35.1
Downhill	118	11.05	< 60 km/h	270	25.3
Up-curve	30	2.8	**Driver condition**		
Down-curve	205	19.2	Fatigue/impaired	272	25.5
Radius < 500 m	175	16.4	Unfamiliar	192	18.0
Radius [500–1000]m	158	14.8	Normal	604	56.5
Radius > 1000 m	64	6.0	**Road category**		
Grade ≥ 4%	199	18.6	Freeway	307	28.7
Grade [2%,4%]	226	21.2	1st-class highway	187	17.5
Straight segment	545	51.1	2nd-class highway	418	39.2
Intersection	121	11.3	Lower lever road	125	11.7
Bridge/tunnel	84	7.9	Urban road	31	2.9
Ramp/interchange	60	5.6	**Vehicle condition**		
**Crash type**			Loss of control	208	19.5
Head-on	353	33.1	Tire/brake failure	291	27.2
Rear-end	284	26.9	Fire occurrence	75	7.0
Angle	134	12.54	Overweight	275	25.7
Sideswipe	21	2.0	Unknown	225	21.1
Hit an object	126	11.8	**Traffic condition**		
Skidding	196	18.4	Free flow	309	28.9
Rollover/overturn	307	28.7	Stable flow	217	20.31
Falling/immersion	147	13.8	Congestion	127	11.9
**Lanes number**			Oversaturation	107	10.0
Lanes num ≤ 2	532	49.8	Unknown	308	28.8
Lanes num > 2	536	50.2			

## 4. Methodology

### 4.1 Association rule mining

An association rule approach is applied to analyze the occurrence and severity of fatal crashes considering different factors and their combinations. The association discovery is an advanced and descriptive data mining tool with a rule-based framework for analyzing valuable interactions between the variables in a database [34]. Compared to existing approaches, ARM does not need to verify the predetermined hypothesis, demonstrates great robustness in extracting information, and can cope with vacant data. This method is recognized as an effective tool for the multi-factor analysis of crash causation owing to its flexibility in exploring hidden relationships [13].

In this study, the Apriori algorithm, the most widely used ARM technique, is applied to investigate the crash data [35]. This algorithm utilizes a bottom-up hash tree structure with level-wise search to mine frequent item-sets from data, which ensures the computational efficiency. An association rule is extracted in the form *X*→*Y*, where *X* and *Y* are disjoint item-sets; *X* is called the antecedent (left-hand-side, LHS), consisting of single or multiple items, and Y is called the consequent (right-hand-side, RHS), containing only one item. In the Apriori algorithm, multiple criteria with predefined threshold values are used to identify frequent item-sets. The extraction and evaluation of rules are mainly based on several parameters, namely support, confidence, and lift.

The support of a rule measures the percentage of antecedents and consequents that occur together in the entire dataset, which can be calculated as

$$\mathrm{Supp}(X\to Y)=P(X\cap Y)=\frac{\#(X\cup Y)}{N};\;\mathrm{Supp}(X)=\frac{\#(X)}{N}$$ (1)

where *N* is the total number of crash records, and #(*X*∪*Y*) is the co-occurrence of item-sets *X* and *Y*.

Confidence indicates the probability that an item *Y* appears simultaneously when the set of *X* factors appears, *P*(*X*|*Y*), which can be interpreted as the credibility of the association rule. This can be calculated as

$$\mathrm{Conf}(X\to Y)=\frac{\mathrm{Supp}(X\to Y)}{\mathrm{Supp}(X)}=\frac{P(X\cup Y)}{P(X)}$$ (2)

Support and confidence represent the probability for the co-occurrence of items covered by the association rule, but directly reflecting the association strength between the LHS and RHS items is difficult. In contrast, lift value links the occurrence of items in the association rule to the expected occurrence under conditional independence [28]. It indicates the probability for the occurrence of RHS changes in the presence of LHS and can be calculated as

$$\mathrm{Lift}(X\to Y)=\frac{\mathrm{Conf}(X\to Y)}{\mathrm{Supp}(Y)}=\frac{\mathrm{Supp}(X\cup Y)}{\mathrm{Supp}(X)∙\mathrm{Supp}(Y)}$$ (3)

When the lift of a rule is higher than one, a significant association exists between the LHS and RHS items. A lift smaller than one indicates two mutually exclusive item-sets, and a lift equal to one indicates that item-sets *X* and *Y* are independent of each other. Therefore, a higher lift is assumed to imply a stronger association with the rule.

The rules used for the analysis shall preferably have a higher support, larger confidence level, and greater lift. Therefore, the minimum thresholds of support (S), confidence (C), and lift (L) are required to be prone to uninteresting rules. The thresholds of support and confidence are usually selected between 1–20% and 30–80%, respectively, and require a lift higher than 1.2 [25]. Most existing studies are mainly based on subjective selection, considering the quantity and quality of filtering rules to define these predefined values [22,23,28,34]. This is because higher indicators may lead to numerous redundant sets, while lower indicators do not adequately investigate hidden associations. Therefore, the trial-and-error approach is applied to determine relatively fair thresholds [25]. We first identify two sets of minimum support and confidence values as 2% and 50%, and 10% and 80%, respectively. When using the first set (2% support, and 50% confidence), the Apriori algorithm generates 63,974 rules, with the lift values distributed from 0.3–5.2. The second set generates only 26 rules with a lift ranging from 0.99 to 1.02. Obviously, neither set of rules can provide meaningful information to form typical patterns of multi-fatality crashes. To better achieve high-associated rules, we continuously iterate the combination of support and confidence probabilities and determine a set of relatively high thresholds as (S ≥ 6%, C ≥ 75%, and L > 1.3) for guaranteeing the credibility of the association rules. The minimum 6% support implies that no rules will be considered effective if they cannot satisfy appearing in at least 64 severe crashes (20% of the total 303 crashes). It is worth noting that the predefined threshold values also directly influence the processing of the Apriori algorithm, and appropriate thresholds effectively ensure the computational efficiency of the overall framework in this study.

### 4.2 Removal of redundant rules

After the generation of rules with thresholds, redundant or invalid rules still exist in the rule set. Furthermore, this study adopts another method to remove redundant rules and ensure the comprehensiveness of the analysis as follows:

1. The lift increase (LIC) tests are performed to ensure that the additional item in rules results in an increase in rules interest [26]. If a rule with n+1 items does not lead to a LIC compared to that for the base rule of n items, the rule is considered redundant. Eq 4 represents the LIC of a rule as
$$\mathrm{LIC}(X_{n+1}\to Y)=\frac{\mathrm{Lift}(X_{n+1}\to Y)}{\mathrm{Lift}(X_{n}\to Y)}$$ (4)
  where *X*_*n+1*_ and *X*_*n*_ are the LHSs of the rules with n+1 and n items, respectively; moreover, *X*_*n*_ ⊆ *X*_*n+1*_. Rules with more items are selected over simpler ones if the LIC condition satisfies the minimum threshold of 1.03.
2. Significance tests are conducted to ensure the generalizability of the filtered rules. Hahsler and Hornik indicated that the confidence of a rule is systematically affected by the frequency of LHS items [36]. Therefore, the selected rules are further verified if the one-sided Fisher’s exact test reaches the expected p-value, which is corrected for multiple tests using the Bonferroni correction [37]. The null hypothesis of the test is that no association exists between the antecedents and consequents. Rules are considered significant if the results reach a significance level of p < 0.01; otherwise, they are excluded.
3. The selected rules are ensured to be practically meaningful for analysis. Some rules with high support may occur frequently in the dataset; however, such rules may not provide any valuable information for safety analysis, e.g., the rule “time: 13:00–17:00 → Light: daylight” or “Limit speed > 80 km/h → Road category: freeway”. Such rules with strong causality but lacking importance are excluded by comparing the relationship between the LHS and RHS items.

### 4.3 Rules graph structures construction

Association rules essentially reflect the potential links of information or probabilities within the dataset. Such interactive associations can be directly indicated by a graphic network, similar to a tree-based structure or Bayesian network [20]. Graph network theory and analysis is a mathematical method for exploring the interrelations between multiple factors. The external observations and internal relations of the rules can be accurately identified based on a network topology with nodes and links. Several scale-free graphical features can be generated to reflect the importance of the factors in rule sets, which compensates for the inadequacies of typical rule tools in single-factor ranking. For example, Weng et al. applied the degree distribution of a rules graph to evaluate the importance of factors in work-zone crashes [13].

In this study, graph theory is used to construct RGSs from the rule set. In simple terms, each factor in the rule set is cast as a node, and the link between any two factors is cast as directed edges to represent the associations between them. In the form of weighted structures, the centrality of nodes can be calculated using graph properties to reflect the importance of each factor in the rule set. Following related studies [13,38,39], the graph properties selected in this study are as follows:

1. Degree represents the number of edges connected to a node, also reflecting the adjacent nodes. The nodes with more connected links are generally considered more valuable in a directed graph. For a rules network, the in-degree and out-degree, which denote the number of pointed-in and pointed-out links, respectively, characterize the frequency of factors in the RHS and LHS for all rules. These can be used to describe the connected relationships within crash factors.
2. Closeness indicates the average distance between a node and other nodes, calculated as the reciprocal of the sum of the lengths of the shortest paths between the node and all other nodes in a graph [22]. Clearly, the higher the value of closeness, the stronger is the centrality of the node. This can be normalized as
$$C(x)=\frac{N}{\Sigma_{y}d(y,x)}$$ (5)
  where *d*(*y*,*x*) is the distance between nodes x and y and N is the number of nodes in the graph. For the RGSs in this study, the closeness of a node can be used as the ranking for the contributions from different factors.
3. PageRank is a measure of node importance and centrality, which was initially an algorithm used to rank webpages [40]. Due to its applicability in directed networks, it has been widely used in other fields, such as social network analysis and traffic demand prediction. The key idea of the algorithm is that the importance of a node depends not only on its centrality but also on the connectivity of the neighboring nodes weighted by out-degree [39]. The PageRank of node X is calculated as
$$\mathrm{PR}(A)=(1‐d)+d(\frac{\mathrm{PR}(V_{1})}{O(V_{1})}+\cdots +\frac{\mathrm{PR}(V_{n})}{O(V_{n})})$$ (6)
  where *V*_*n*_ is the n_th_ adjacent node of node *A*, *O*(*V*_*n*_) is the out-degree of node *V*_*n*_, and parameter *d* is a damping factor (set to 0.85). In Eq (6), PageRank is defined recursively and can be calculated using an iterative algorithm. In an RGS, PageRank can identify key factors that are closely associated with other factors.
4. Modularity reflects the clustering relationships between nodes within the graph structure. Through hierarchical division, a complex network can be divided into a number of subordinate modules or communities, which demonstrates the potential structural composition within the network [41]. The nodes in the same module are more closely related and can be regarded as having similar attributes. The detection of modularity structure is based on the hierarchical agglomeration algorithm proposed by Blondel, and the modularity of the network can be measured using the modularity coefficient Q [42]. The modularity Q is defined as
$$Q=\frac{1}{2m}\sum_{\mathrm{vw}}\left[A_{\mathrm{vw}}‐\frac{k_{v}k_{\mathrm{vw}}}{2m}]\delta (c_{v},c_{w}\right)$$ (7)
  where m is the number of modules; v and w are the vertexes of the network; *k*_*i*_ is the degree of vertex i; *A*_*vw*_ is an element of the adjacency matrix of the network, and if v and w are connected, *A*_*vw*_ = 1, otherwise *A*_*vw*_ = 0; *c*_*i*_ is the module to which vertex i belongs; the *δ* function *δ*(*i*,*j*) is 1 if i = j and 0 otherwise. The forms of different modularity structures can be compared by the coefficient Q. In practice, a value of Q higher than 0.3 indicates a significant community structure of the network. The rules and factors within the same module imply similar crash characteristics and are used as references for grouping rules in this study.

## 5. Results and analysis

After the redundancy elimination and significance tests, 1452 rules are extracted from 2917 rules that met the minimum thresholds. A two-step analysis is then conducted. First, the RGS is constructed to verify the crash patterns and key factors of multi-fatality crashes. Second, the rules involving high-frequency RHS items and rules with high-value indicators are further analyzed to explore the hidden interactions between different contributory factors.

### 5.1 Analysis of RGSs

**Fig 2:**
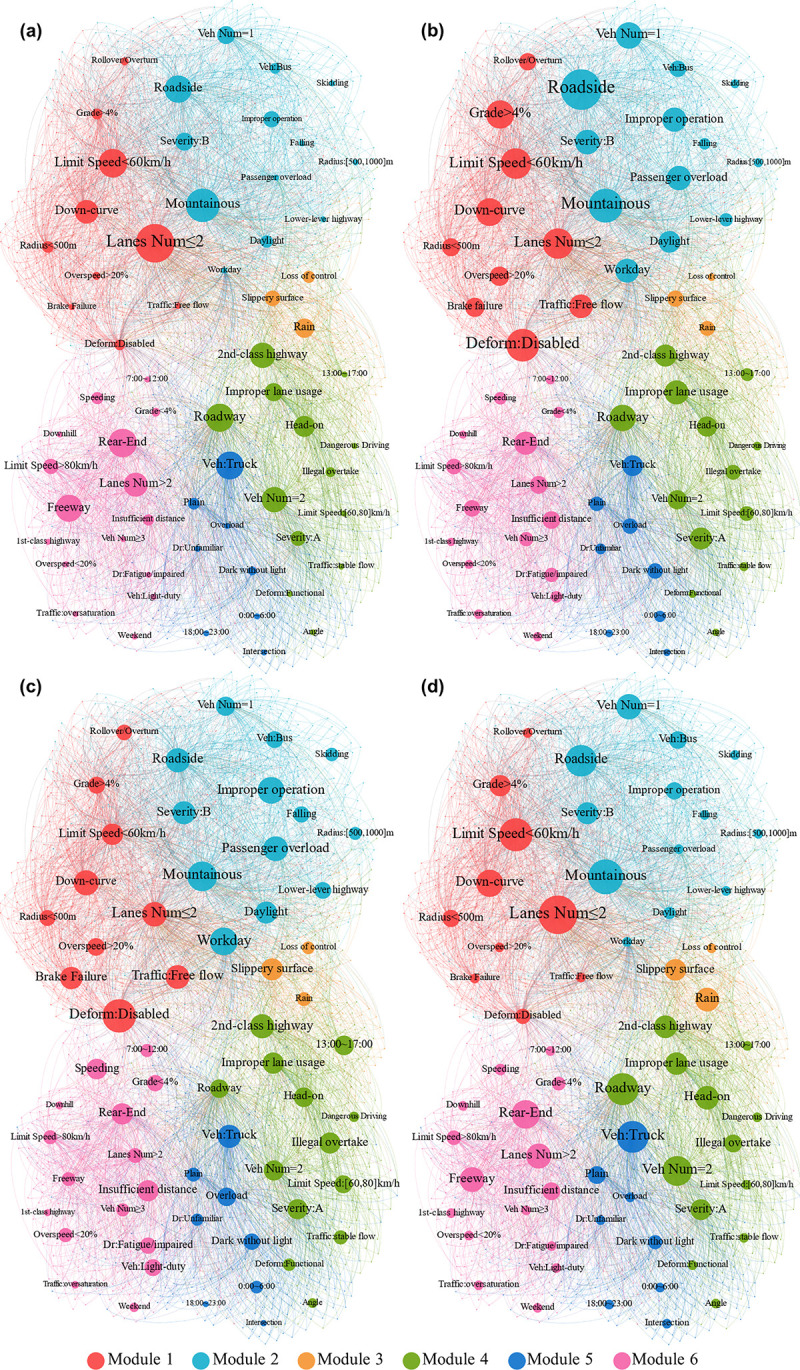
Modular RGS based on different graph properties. (a) In-degree, (b) Out-degree, (c) Betweenness, and (d) PageRank.

Based on the generated rule sets, the Gephi interactive network visualization platform is used to create four directed rule network structures according to different graph structure properties, as shown in Fig 2. Each graphic structure contains 1515 nodes and 5340 edges, of which 63 nodes represent crash factors occurring in the rules, with sizes proportional to the node properties. The remaining 1452 nodes represent independent rules connecting the LHS and RHS items. The RGSs are internally clustered into six modules, which are distinguished by different colors in Fig 2. The modular structure is generated by the Force Atlas algorithm built into the Gephi platform, which guarantees the computational efficiency of the framework. The optimal 6-mod structure obtained the outperformed modularity coefficient Q of 0.441 > 0.3 compared to other structures. As mentioned above, modularity is used to measure the strength of the network division. This means that nodes of the same color are tightly associated, while other nodes of different modules are sparsely linked. The correlations between different crash-related factors can be intuitively reflected in the graph-based visualization.

Fig 2(A) and 2(B) represent the in-degree and out-degree of different nodes in the network, which represent the frequency of the factors in the RHS and LHS, respectively. The higher the in-degree, the more rules that point to the node. Note that “Lanes num ≤ 2, Location: roadside, Terrain: mountainous, and Vehicle type: truck” are the most common backgrounds for fatal crashes. Moreover, the out-degree indicates the co-occurrence of node characteristics and specific crash types. Factors involving lower geometric design, risky driving behavior, and violations showcase higher values, meaning that these variables decisively lead to serious causalities.

Fig 2(C) and 2(D) show the closeness and PageRank for different variables. Although both indicators reflect the connectivity and centrality of nodes, their distribution characteristics are not the same. The value of closeness reflects the comprehensive node strength such that larger nodes gather at the center of Fig 2(C). Correspondingly, the larger nodes in Fig 2(D) are generally scattered in different modules (such as "Rain" in module 3 and "Rear-end" in module 6). This implies that nodes with a high PageRank play a pivotal role in their modules.

As a hierarchical framework of severe crashes, Table 2 lists the contribution of the top eight individual factors to the network structure under a system-level typology. The indicators are divided into five high-level constructs and ranked based on the closeness of the nodes. The most critical nodes are “Improper operation,” “Passenger overload,” “Lanes num ≤ 2,” “Terrain: mountainous,” and “Location: roadside” for driver, vehicle, roadway, environment, and crash factors, respectively. This finding is consistent with the results of previous studies [12,33].

**Table 2:** Contributions of factors to the graph structures based on closeness.

**Driver factors**	**Contribution (%)**	**Roadway factors**	**Contribution (%)**
Improper operation	22.24	Lanes num ≤ 2	19.23
Improper lane usage	19.41	2nd-class highway	14.83
Illegal overtaking	15.31	Grade > 4%	14.45
Speeding	11.96	Down-curve	13.82
Insufficient distance	9.91	Radius < 500 m	12.38
Dr: fatigue/impaired	8.85	Lanes num > 2	9.24
Dr: unfamiliar	7.43	Lower-lever highway	8.63
Dangerous driving	4.89	Freeway	7.42
**Environment factors**	**Contribution (%)**	**Vehicle factors**	**Contribution (%)**
Terrain: mountainous	20.04	Passenger overload	17.31
Traffic: free flow	17.71	Veh type: truck	17.18
Surface: wet/slippery	16.11	Tire/brake failure	15.91
Light: daylight	16.03	Overweight	12.59
Weather: rain	7.84	Overspeed > 20%	11.88
Light: dark without light	7.65	Veh type: bus	10.19
Traffic: stable flow	7.54	Veh type: light-duty	9.87
Terrain: plain	7.08	Loss of control	5.07
**Crash factors**	**Contribution (%)**		
Location: roadside	19.77		
Location: roadway	17.82		
Crash: head-on	15.66		
Crash: rear-end	13.43		
Crash: rollover/overturn	13.07		
Crash: falling	10.28		
Crash: skidding	7.24		
Crash: angle	2.73		

The graph structure has a high modularity Q of 0.448, which indicates significant homogeneous effects within independent modules, and can be used for clustering the rule set. According to the PageRank in Fig 2(D), variables with the same color tend to have similar features:

1. Modules 1 and 2 are spatially close within the graph structure and represent the patterns of mountain and ROR crashes, respectively.
2. Module 3 is relatively small in scale and represents the crash type caused by loss of control.
3. Modules 4 and 6 indicate the patterns of head-on and rear-end crashes with high-value factors of {2nd-class highway, Head-on, Improper lane usage} and {Freeway, Rear-end, Lanes num > 2}, respectively.
4. Module 5 primarily reflects the casualties caused by truck overloading on plain highways.

The differences between the modules are also reflected in the graphical properties of the internal nodes. Modules 1 and 2 include fewer crash factors with a higher mean value of node properties, indicating the homogeneous effects of aggregated elements within mountainous crash patterns. In contrast, modules 4 and 6 contain approximately half of the factors, but the strength of each node is low, which demonstrates the complexity and diversity of accident causation for rear-end and head-on patterns. The representative rules are explained in detail along with the modules in the following sections.

### 5.2 General analysis of association rules

**Fig 3:**
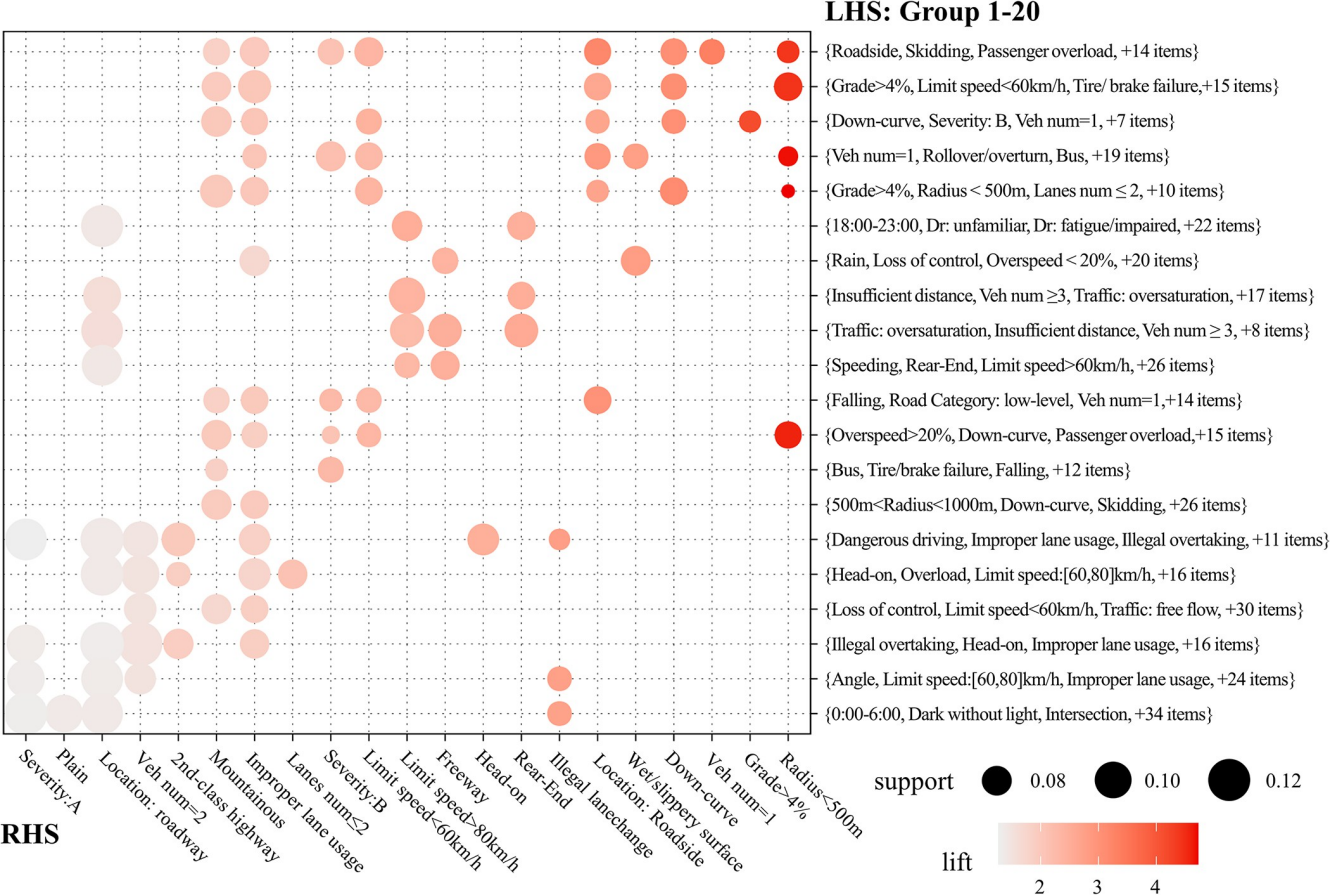
Group matrix-based visualization of 1452 rules.

RGSs indicate the relationships among characteristics, but clarifying the association between the antecedents and consequents is difficult. In this study, a group of matrix-based visualizations is performed to refine the association. The rule set is divided into 20 groups using the K-means clustering tool. Fig 3 presents a balloon plot to visualize the relations between the representatives of grouped antecedents and the consequents of all 1452 rules. A large circle size indicates a high support value, and the darker the color, the higher is the lift value. In addition, the bubbles in the figure are reordered such that the aggregated lift value decreases from the top left to the bottom right. Note that the variables contained in the high-lift and high-support groups are different from each other.

The factors involved in the high-lift groups (top-right corner of Fig 3) are almost present in modules 1 and 2. Improper operation, vehicle-related defects, low radius, and crash dynamic factors (skidding, rollover, and ROR) significantly increased the lift of single-vehicle crashes on mountain roads. Correspondingly, groups with high support represent frequent item-sets in severe crashes, which are mostly composed of driver errors and environmental conditions. The representative group with the highest support is {0:00–6:00, Dark without light, Speeding}, which corresponds to RHS items, including plain terrain, on-road crashes, and illegal overtaking. The preliminary results show that serious fatal crashes are more likely to be caused by poor lighting conditions, and dangerous driving aggravates the casualty risk in plain areas.

**Fig 4:**
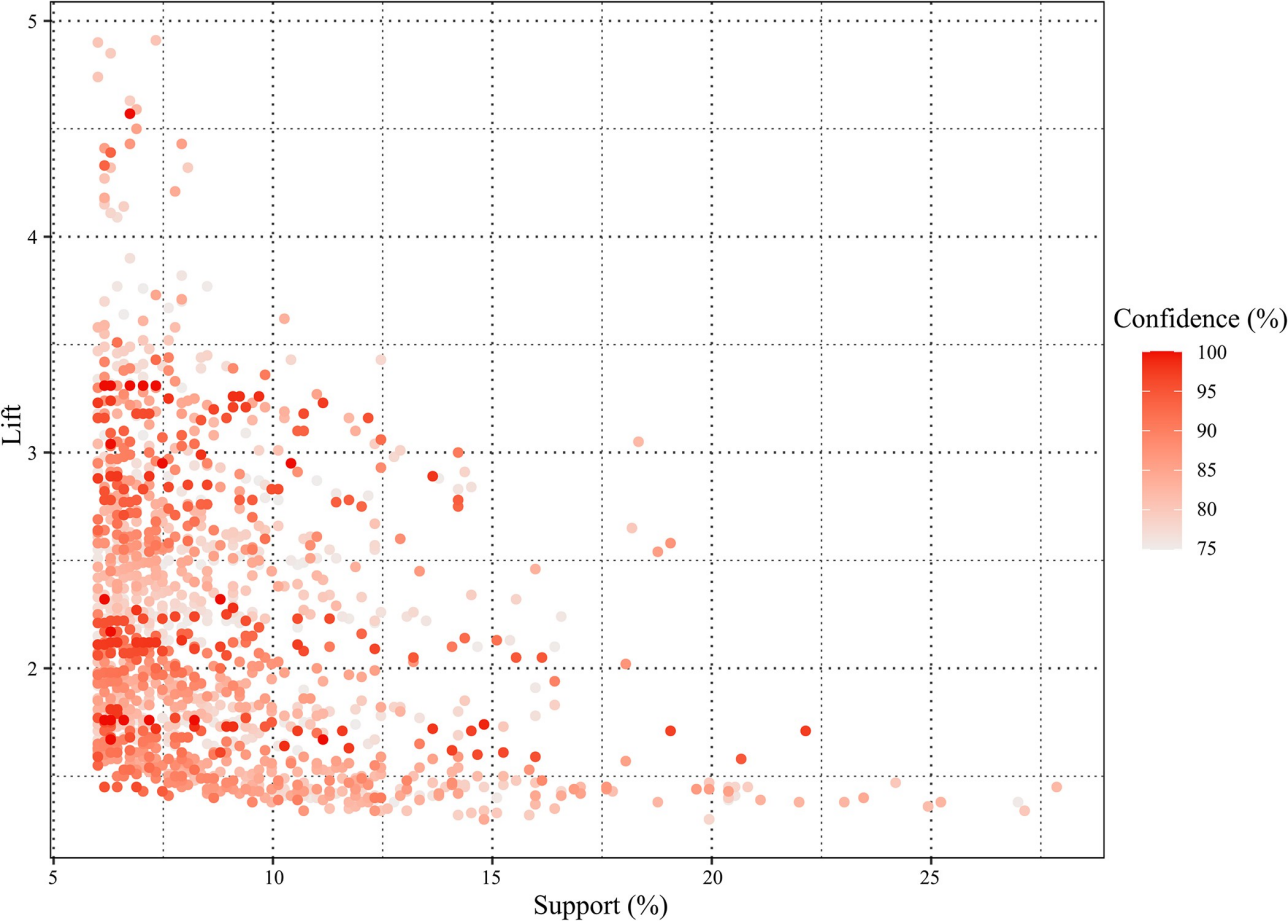
Distribution of lift, support, and confidence.

Fig 4 visualizes the relationship between the lift, support, and confidence levels of the 1452 rules. Each scatter represents an association rule, and its color intensity reflects confidence. The support of 75.04% of rules is between 6% and 10%, and 70.16% of rules have a confidence value over 80%, which ensures the credibility of the rule set. According to Hong et al., all refined rules in this study are considered high promotion rules (range from 1.30 to 4.91), and strong association rules (lift > 2) account for more than 50% of rules. The lift of rules shows a certain inverse relationship with the support, and the rules with a higher lift tend to show lower support, which is consistent with the results of the group matrix in Fig 3. The separated relationship between the high lift and high support groups inspires to further explore the differences and connections among high-value rules.

### 5.3 High-value rules

Table 3 lists the 45 high-interest rules extracted according to lift, support, and confidence in decreasing order as well as the LIC and module attributes of these rules. LIC items are highlighted in bold thereby emphasizing the key role of specific items. The high-value rules based on different indicators exhibit structural differences with modularity clustering. All high lift rules point to mountainous features represented by modules 1 and 2, whereas high support rules appear only in modules 4, 5, and 6, which are dominated by driver-related factors.

**Table 3:** Rules sorted by top indicator (lift, support, and confidence).

No	LHS	RHS	S(%)	C(%)	L	LIC	Mod
***Top lift rules***							
a1	Down-curve, Lanes num ≤ 2, **Traffic: free flow**^a^	Radius<500m	7.33	80.65	4.91	1.24	1
a2	Down-curve, Limit speed<60km/h, **Traffic: free flow**	Radius<500m	6.01	80.39	4.90	1.19	1
a3	Grade > 4%, Lanes num ≤ 2, **Traffic: free flow**	Radius<500m	6.30	79.63	4.85	1.19	1
a4	Down-curve, **Improper operations**, Lanes num ≤ 2	Grade > 4%	6.01	80.39	4.74	1.34	1
a5	Down-curve, Rollover/overturn, **Limit speed<60 km/h**	Grade > 4%	6.74	79.31	4.63	1.30	1
a6	Down-curve, Rollover/overturn, **Lanes num ≤ 2**	Grade > 4%	6.89	82.46	4.59	1.29	1
a7	Down-curve, Location: roadside, **Improper operations**	Veh num = 1	6.74	100	4.57	1.43	2
a8	Mountainous, Location: roadside, **Passenger overload**	Veh num = 1	6.89	85.45	4.50	1.47	2
a9	Veh: bus, Mountainous, **Improper operations**	Veh num = 1	6.74	86.79	4.43	1.80	2
a10	Location: roadside, **Improper operations**, Traffic: free flow	Veh num = 1	7.92	85.71	4.43	1.35	2
a11	Location: roadside, **Passenger overload,** Limit speed < 60 km/h	Veh num = 1	6.16	85.71	4.41	1.45	2
a12	Radius < 500 m, Limit speed < 60 km/h, **Tire/brake failure**	Down-curve	6.30	93.48	4.39	1.37	1
a13	Grade > 4%, **Rollover/overturn**, Limit speed < 60 km/h	Down-curve	6.16	93.33	4.33	1.37	1
a14	**Down-curve**, Limit speed < 60 km/h, Tire/brake failure	Grade > 4%	8.06	79.39	4.32	1.51	1
a15	**Veh num = 1**, Radius < 500 m	Grade > 4%	6.30	81.13	4.32	1.27	1
***Top support rules***							
a16	Crash: rear-end	Lanes num>2	27.86	82.25	1.45	/	6
a17	Crash: rear-end	Roadway	27.13	80.09	1.34	/	6
a18	Improper lane usage	Veh num = 2	26.98	75.41	1.38	/	4
a19	Veh: truck, **Terrain: plain**	Roadway	25.22	82.69	1.38	1.10	5
a20	Veh: truck, **Lanes num > 2**	Roadway	24.93	81.73	1.36	1.09	4
a21	Crash: head-on	Veh num = 2	24.19	80.10	1.47	/	4
a22	Severity: A, Veh: truck, **Veh num = 2**	Roadway	23.46	84.21	1.40	1.06	4
a23	Crash: rear-End, **Freeway**	Lanes num>2	22.14	97.42	1.71	1.18	6
a24	Severity: A, **Improper lane usage**	Roadway	21.99	82.87	1.38	1.15	4
a25	Location: roadway, **Improper lane usage**	Veh num = 2	20.82	79.33	1.45	1.19	4
a26	Insufficient distance	Roadway	20.67	94.63	1.58	/	6
a27	Location: roadway, **Improper lane usage**	Veh: truck	20.53	78.21	1.45	1.16	5
a28	Deform: disabled, **Improper lane usage**	Veh: truck	20.53	76.09	1.41	1.29	5
a29	Veh: truck, **Crash: rear-end**	Roadway	20.38	85.80	1.43	1.14	6
a30	Speeding	Lanes num>2	20.38	79.43	1.40	/	6
***Top confidence rules***							
a31	**Mountainous**, Crash: falling	Roadside	7.77	100	3.31	1.12	2
a32	Deform: disabled, Crash: falling, **Lanes num ≤ 2**	Roadside	7.77	100	3.31	1.06	2
a33	Veh num = 1, **Passenger overload**, Lanes num ≤ 2	Roadside	7.62	100	3.31	1.07	2
a34	Down-curve, Passenger overload, **Improper operations**	Mountainous	7.33	100	3.31	1.07	2
a35	Veh num = 1, **Road category: low-level highway**	Roadside	6.60	100	3.31	1.25	2
a36	Mountainous, Radius<500 m, **Traffic: free flow**	Lanes num≤2	8.80	100	2.32	1.07	1
a37	Down-curve, **Location: roadside**, Improper operations	Veh num = 1	6.74	100	4.57	1.35	2
a38	Crash: rear-end, **Speeding**, Limit speed>80 km/h	Lanes num>2	8.21	100	1.76	1.13	6
a39	Illegal overtake, **Limit speed>80 km/h**, Freeway	Lanes num>2	7.18	100	1.76	1.07	6
a40	Veh: truck, **Surf: wet/slippery**, Freeway	Lanes num>2	6.74	100	1.76	1.07	6
a41	Speeding, Limit speed>80km/h, **Dr: fatigue/impaired**	Lanes num>2	6.30	100	1.76	1.26	6
a42	Veh num ≥ 3, **Insufficient distance**, Freeway	Rear-end	7.48	100	2.95	1.23	6
a43	Veh: truck, **Insufficient distance**, Freeway	Rear-end	10.41	100	2.95	1.20	6
a44	**Insufficient distance**, Limit speed>80km/h, Freeway	Roadway	11.14	100	1.67	1.53	6
a45	**Dark without light**, Deform: disabled, Insufficient distance	Roadway	6.30	100	1.77	1.09	6

^a^ LIC items are highlighted in bold.

Higher lift values indicate stronger associations between the LHS and RHS items. The high lift rules in Table 3 involve 14 sub-items. In addition to geometric design factors, the sub-items include the traffic environment (free flow and limit speed < 60 km/h), vehicle elements (bus involved, brake failure, and passenger overload), and driver factors (improper operations). According to rules a1 to a7 in Table 3, item-set {Down-curve, Rollover, Improper operations} represents the most significant crash patterns in mountainous areas. A small curve radius and critical gradient significantly increase the odds of serious casualties in mountainous areas (lift > 4.32), which is consistent with previous studies [27]. Moreover, the risk of single-vehicle crashes is significantly increased with the item-set {Improper operations, Passenger overload, Roadside, Traffic: free flow} (rules a7 to a11). This combination of factors increases the probability of single-vehicle crashes by a lift of 4.41 and a confidence level of 85.71%. In addition, LIC items, such as overturn and brake failure, increase the lift of the down-curve rules by approximately 20%, implying that vehicle defects and improper driving maneuvers significantly increase the driving risk in mountainous roadways.

Higher support reflects a higher proportion of the dataset and represents the most common patterns in multi-fatality crashes. Most high support rules are associated with double-vehicle rear-end or head-on collisions on the roadway. In contrast to the co-occurrence of geometric design factors in the top lift rules, behavioral factors, such as insufficient distance, improper lane usage, and speeding, typically appear as antecedents in the high support groups and are strongly associated with truck crashes and plain terrain (rules a18 and a24–a30). These rules account for more than 20% of actual cases, indicating that illegal operations are the most common cause of serious fatal crashes, which is consistent with the results reported by Xu et al. [33].

Notably, the top confidence rules in Table 3 all have a 100% confidence level, which implies that the antecedent term is a sufficient condition for the consequent term [13]. Poor lighting conditions, slippery surfaces, and violations significantly increase the probability of serious rear-end crashes on high limit speed highways (rules a38–a45, lift > 1.67), whereas illegal overcrowding greatly increases the probability of ROR crashes (rule a33, lift = 3.31). Another interesting finding is that, as the subrule of rule a30, the LIC of rule a41 reaches 1.25, implying a more vital interdependence between fatigue driving and overspeed. Traffic agencies should focus on high-value rules to implement more effective safety measures.

### 5.4 Association rules involving high-frequency RHS items

**Fig 5:**
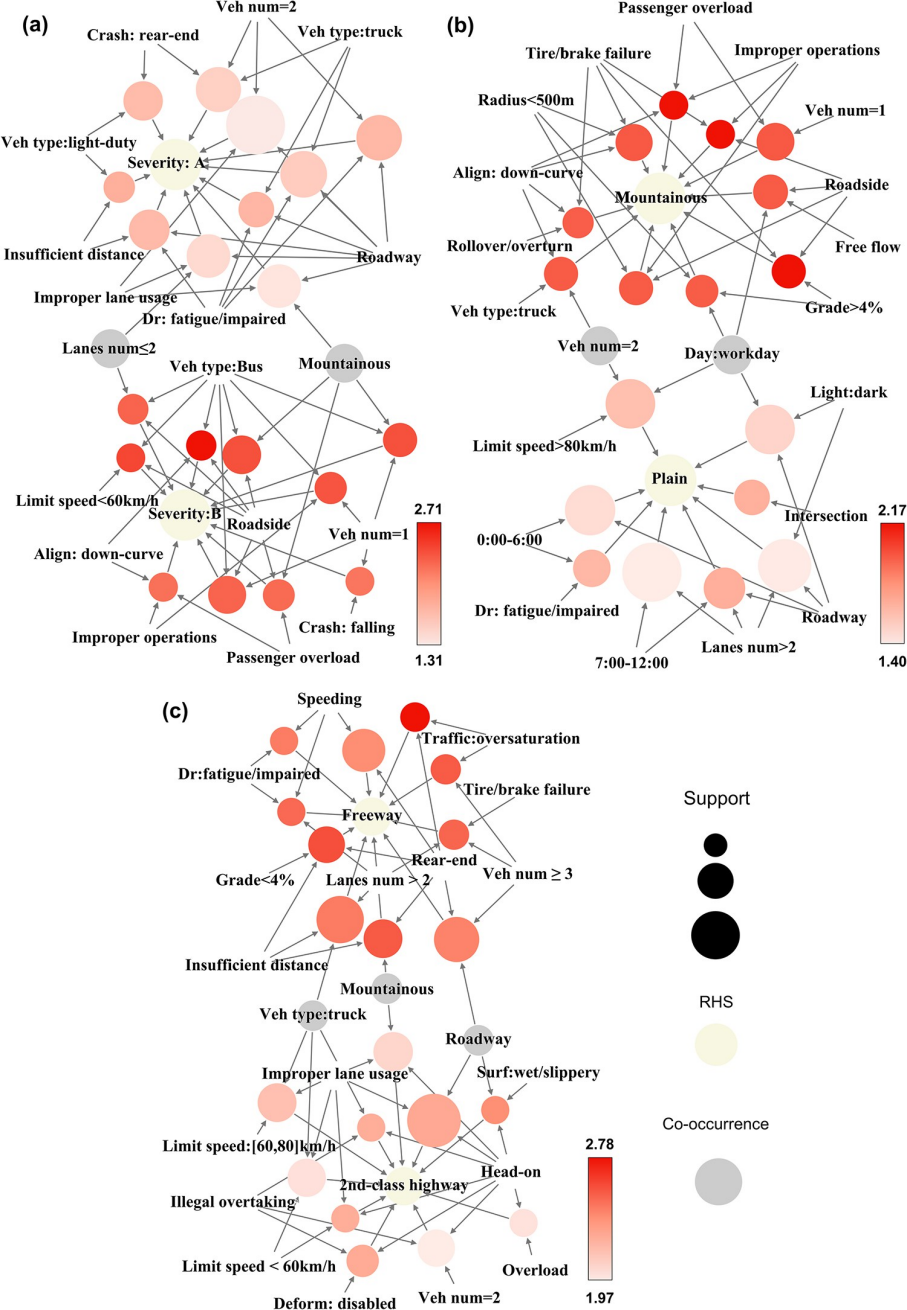
Graph structure for specific rule set. (a) Severity: A & Severity: B, (b) Mountainous & Plain (terrain), and (c) Freeway & 2nd-class highway (road category).

To further explore the potential patterns of multi-fatality crashes, high-frequency RHS item-sets with mutually exclusive properties are extracted (Table 4), namely {Severity: A} & {Severity: B}, {Terrain: mountainous} & {Terrain: plain}, and {Road category: freeway} & {Road category: 2nd-class highway}. These paired RHS items belong to the high-frequency items in Table 1 and correspond to separated modules with different crash patterns. The characteristics of the rules are visualized by graph structures in Fig 5 to demonstrate the interactions in each LHS and RHS. The labels in the graphs represent crash factors, and the nodes represent different independent rules. Their color and size reflect the support and lift of the rules, the same as shown in Fig 2. The following subsections analyze these specific rule sets in terms of incident characteristics and rule values.

**Table 4:** Rules involving high-frequency RHS items.

No	RHS	LHS	S (%)	C(%)	L
b1	Severity: A	Veh type: light-duty, Insufficient distance	7.04	92.31	1.43
b2		Veh type: truck, Roadway, Dr: fatigue/impaired	8.65	90.77	1.40
b3		Roadway, Veh num = 2, Dr: fatigue/impaired	12.46	90.43	1.40
b4		Roadway, Insufficient distance, Dr: fatigue/impaired	10.56	90.00	1.39
b5		Veh type: light-duty, Crash: rear-end	10.12	89.61	1.39
b6		Veh type: truck, Roadway, Dr: fatigue/impaired	12.90	87.13	1.35
b7		Veh type: truck, Veh num = 2, Crash: rear-end	12.32	86.60	1.34
b8		Roadway, Improper lane usage, Lanes num ≤ 2	11.73	85.11	1.33
b9		Terrain: mountainous, Improper lane usage, Roadway	9.73	86.90	1.32
b10		Veh num = 2, Roadway, Improper lane usage	17.60	84.51	1.31
b11	Severity: B	Veh type: bus, Down-curve, Roadside	6.60	95.74	2.71
b12		Veh type: bus, Roadside, Limit speed < 60 km/h	6.01	93.18	2.64
b13		Veh type: bus, Terrain: mountainous, Roadside	6.60	91.84	2.60
b14		Veh type: bus, Veh num = 1, Terrain: mountainous	9.82	91.78	2.59
b15		Veh type: bus, Veh num = 1, Improper operations	8.06	91.67	2.57
b16		Veh type: bus, Veh num = 1, Roadside	7.62	91.23	2.45
b17		Veh type: bus, Roadside, Lanes num ≤ 2	7.33	90.91	2.45
b18		Terrain: mountainous, Roadside, Passenger overload	9.53	90.28	2.37
b19		Down-curve, Improper operations, Passenger overload	6.60	90.00	2.36
b20		Veh num = 1, Crash: falling	13.34	86.67	2.35
b21	Terrain: mountainous	Down-curve, Improper operations, Passenger overload	6.01	100.0	2.17
b22		Improper operations, Roadside, Tire/brake failure	6.01	98.18	2.17
b23		Grade > 4%, Tire/brake failure, Roadside	7.04	98.04	2.17
b24		Roadside, Day: workday, Free flow	7.18	98.00	2.12
b25		Radius < 500 m, Roadside, Improper operations	7.04	97.96	2.12
b26		Veh type: truck, Veh num = 2, Down-curve	7.04	97.92	2.12
b27		Radius < 500 m, Grade > 4%, Day: workday	6.74	97.78	2.12
b28		Down-curve, Rollover/overturn, Tire/brake failure	6.45	97.73	2.12
b29		Veh num = 1, Passenger overload	7.77	97.62	2.12
b30		Down-curve, Radius < 500 m, Tire/brake failure	7.62	97.30	2.12
b31	Terrain: plain	Time: 7:00–12:00, Roadway, Lanes num > 2	8.50	84.06	1.56
b32		Intersection	7.33	83.33	1.55
b33		Dr: fatigue/impaired, Time: 0:00–6:00,	7.62	81.25	1.51
b34		Veh num = 2, Day: workday, Limit speed > 80 km/h	10.12	79.31	1.47
b35		Day: workday, Roadway, Light: dark	10.12	76.67	1.42
b36		Time: 0:00–6:00, Roadway	10.26	76.09	1.41
b37		Roadway, Light: dark, Lanes num > 2	10.85	75.51	1.40
b38		Time: 7:00–12:00, Lanes num > 2	12.02	75.23	1.40
b39	Road category:freeway	Crash: rear-end, Traffic: oversaturation,	6.30	95.56	2.78
b40		Grade < 4%, Crash: rear-end, Insufficient distance	7.77	92.98	2.71
b41		Mountainous, Crash: rear-end, Insufficient distance	8.21	91.80	2.68
b42		Traffic: oversaturation, Veh num ≥ 3	6.45	91.67	2.67
b43		Veh num ≥ 3, Lanes num > 2, Tire/brake failure	6.45	89.80	2.62
b44		Speeding, Dr: fatigue/impaired, Lanes num > 2	7.18	89.09	2.60
b45		Veh type: truck, Insufficient distance, Lanes num > 2	10.85	88.10	2.57
b46		Speeding, Dr: fatigue/impaired	7.48	87.93	2.51
b47		Veh num ≥ 3, Crash: rear-end, roadway	6.30	86.00	2.51
b48		Crash: rear-end, Speeding	9.97	83.95	2.45
b49	Road category: 2nd-class highway	Crash: head-on, Roadway, Surf: wet/slippery	6.01	89.13	2.25
b50		Crash: head-on, Roadway, Improper lane usage	12.32	81.55	2.06
b51		Crash: head-on, Deform: disabled, Illegal overtake	6.89	81.03	2.05
b52		Veh type: truck, Crash: Head-on, Illegal overtake	6.01	80.39	2.03
b53		Crash: head-on, Improper lane usage, Limit speed < 60 km/h	6.60	80.39	2.03
b54		Veh type: truck, Improper lane usage, Limit speed [60–80] km/h	8.21	77.78	2.03
b55		Terrain: mountainous, Crash: head-on, Improper lane usage	8.36	76.00	2.01
b56		Crash: head-on, Overload	6.01	75.93	1.98
b57		Veh type: truck, Improper lane usage, Limit speed < 60 km/h	8.18	75.69	1.98
b58		Veh num = 2, Crash: head-on, Illegal overtake	7.92	77.89	

#### 5.4.1 {Severity: A} & {Severity: B}

The rule sets with {Severity: A} and {Severity: B} as the consequents belong to Modules 4 and 2, respectively. Fig 5(A) effectively reflects the patterns of the 20 rules involving different crash severities. The crashes of severity level A are strongly associated with light-duty cars and trucks along with illegal driving behaviors, such as fatigued driving, improper lane usage, and insufficient distance. The main factors involved in severity level B are crash dynamics, including passenger overload, improper operations, single-vehicle, and bus involvement, as well as environmental factors, including mountainous roads, lanes num ≤ 2, limit speed < 60 km, and roadside location. The significant differences between the two sets are the type of vehicle involved (bus vs. truck), crash location (roadside vs. on-roadway), crash type (single vehicle vs. double-vehicle), and driver-related errors.

ROR crashes involving buses significantly affect the over-representation of high-severity crashes with lift values ranging from 2.45 to 2.71 (rules b11–b18). The item-set {Bus, Down-curve, Roadside} has the highest lift and confidence of 2.71 and 95.74%, respectively. This indicates that the proportion of extreme fatalities caused by a bus rushing out of roadside at a downhill curve section is as high as 95.74, which is 2.71 times the average proportion. Compared with the related lower frequency of other rules, rule b20 indicates that 13.34% of crashes in the dataset are caused by single-vehicle falling from roadside, accounting for 37.8% of crashes with severity level B. Moreover, the curve alignment of mountain roads may greatly increase the driving difficulty of overcrowded buses, resulting in leaving the carriageway and a large number of casualties. Relatively poor dynamic conditions and a lack of safety measures are the key causes of single crashes in large vehicles [43,44].

#### 5.4.2{Terrain: mountainous} & {Terrain: plain}

Due to the differences in road alignment and collision dynamics, the mountainous and plain rules of modules 2 and 5, respectively, exhibit completely distinct characteristics. Overall, 18 high-value rules are identified for these two groups with 21 elements, as shown in Fig 5(B). Vehicle brake failure, roadside factors, and down-curve alignment are the most frequent items for mountain terrain, resulting in an average lift value of 2.12 (rules b21–b30). Correspondingly, a higher support of the plain group implies a higher frequency in the dataset. Rules b33–b37 indicate that fatigue driving on high limit speed highways without lighting at night increases the probability of plain crashes by 1.4–1.51 times, which accounts for at least 10% of the crash data.

All rules related to mountainous terrain as RHS have the confidence of over 97.3%, which can be regarded as a sufficient condition for mountain serious accidents. Among them, roadside location, single-vehicle, overcrowding, and improper operations also co-occurred in the {Severity: B} rule set, suggesting that mountain crashes tend to cause more severe fatalities. Undoubtedly, small-radius curves combined with critical slopes considerably increase the risk of driving malfunction and potential vehicle defects (rule b21, lift = 2.17). Additionally, braking failure of vehicles on long and steep downhill has always been the leading cause of significant casualties on Chinese mountainous highways (rules b22 and b23). In addition to roadway reconstruction and safety measures, on-board safe speed for brake temperature is another advanced technology for vehicle risk perception [45].

Notably, although the crashes in plain areas account for 52.90% of all records, there are only eight rules as RHS because most rules cannot satisfy the threshold of lift and confidence. In sharp contrast, the number of rules with {mountain terrain} as a consequent reaches 180, and the least lift value is greater than 1.6. Therefore, generalizing the patterns of severe crashes in plains at a relatively high confidence level is difficult.

#### 5.4.3. {Freeway} & {2nd-class highway} (road category)

The rules with freeway and 2nd-class highway as consequents are mostly derived from modules 6 and 4. On-roadway location, mountain terrain, and truck crashes are the co-occurrence items of these two groups. According to the frequency in Fig 5(C), the patterns related to freeways include rear-end, multiple-vehicle collision, speeding, insufficient distance, and fatigued driving. Moreover, the characteristics of 2nd-class highways include head-on crashes, driving on the wrong side, truck accidents, and illegal overtaking. The collision type (rear-end and head-on) is the most evident difference due to the full access control of expressways and the non-existence of the median for 2nd-class highways in China.

For 2nd-class highway crashes, the features with the highest lift and confidence are the roadway frontal crashes caused by slippery pavement (rule b49). For this rule, the percentage of 2nd-class highway crashes is 89.13%, and the lift value is greater than 2.25. Slippery pavement, which is related to reduced friction, significantly affects the lane keeping and braking distance of vehicles as well as may directly lead to skidding and opposite collisions [46]. Moreover, inappropriate maneuvers, including illegal overtaking and improper lane usage, are evidently the most contributing driver factors (rules b51–b55, b57, and b58).

In addition to the features mentioned, rules b39 and b42 emphasize the adverse impact of multi-vehicle crashes caused by traffic interruptions on highway traffic safety (lift > 2.67). Unlike periodic congestion, traffic oversaturation is typically caused by traffic emergencies or near-crash events. Sudden blocking causes a sharp capacity drop and seriously affects traffic continuity and safety in bottleneck area [47,48]. Reasonable inducement measures and effective evacuation are critical to reducing such events. Furthermore, rule b43 indicates that vehicles with defective brakes can result in severe fatalities, even on adequately protected freeways.

## 6. Discussion

This study utilized the association rule technique to discover the contributing factors and interdependent characteristics of roadway multi-fatality crashes in China. The RGS for pattern recognition and association visualization is explored in conjunction with graph theory, which facilitates the ranking of factors. From the perspective of methodology, combining the Apriori algorithm with RGS can effectively identify crash patterns and mechanisms previously hidden in crash data.

With the modularity structure in Fig 2, the obtained 1452 rules are divided into six clusters, including ROR crashes, vehicle out-of-control, frontal collisions, and rear-end collisions, as well as the terrain characteristics of mountainous and plain areas. According to Table 3 and the graph structure, modules 1 and 2 represent the crash characteristics in mountainous areas. These two groups include fewer crash factors and showcase stronger node properties with higher lift values, illustrating the interactions among the ROR, single-vehicle, severe fatalities, and mountain crashes. Several crash patterns can be extracted from modules 1 and 2:

1. Road alignment and driving behavior: Mountainous roads on sharp horizontal curves with steep grades are more likely to cause serious fatalities. Moreover, driver maloperation and fatigued driving directly increased the probability of such crashes.
2. Vehicle factors and crash dynamics: Vehicle defects (loss of control or brake failure) are highly related to the overrepresentation of rollover or falling accidents, and illegal overloading aggravated the severity of ROR crashes.
3. Traffic environment and driver factors: Single-vehicle crashes are more likely to occur under free-flow traffic conditions on mountainous roads. This association is even stronger for driver-related errors (e.g., unfamiliarity or fatigue) at high posted speed limits.

Based on the identified patterns of multi-fatality crashes, several novel findings can be concluded in terms of regional differences, crash characteristics, road categories and driving behavior. Compared with the concentrated patterns of mountainous crashes, the causations of plain accidents are more complex and diverse. Modules 4–6 characterize the types represented by two-vehicle or multi-vehicle crashes in plain area, with rear-end and head-on collisions being the most frequent. For different road types, high-class highways with adequate protection measures are more likely to result in severe fatalities with improper maneuvers and unfavorable roadside conditions. In terms of low-level highways, head-on crashes along with improper maneuvers, such as illegal overtaking, driving on the wrong side, and unfamiliar surroundings are the main factors affecting traffic safety. Furthermore, rear-end crashes involving trucks are highly associated with fatigued driving, insufficient following distance, overspeed, and traffic oversaturation. Note that these novel conclusions illustrate higher confidence and represent the most common patterns for severe crashes.

Additionally, the analysis of high-value rules leads to an interesting conclusion that rules combining drivers, road environment, and crash factors tend to have higher confidence and lift. For example, the combination of curve alignment (road environment), roadside location (crash dynamic), and improper operation (driver behavior) is strongly correlated with single-vehicle crashes (confidence = 100% and lift = 4.57). This finding also demonstrates that the hidden associations between various factors contribute to the frequency and severity of multi-fatality crashes.

**Fig 6:**
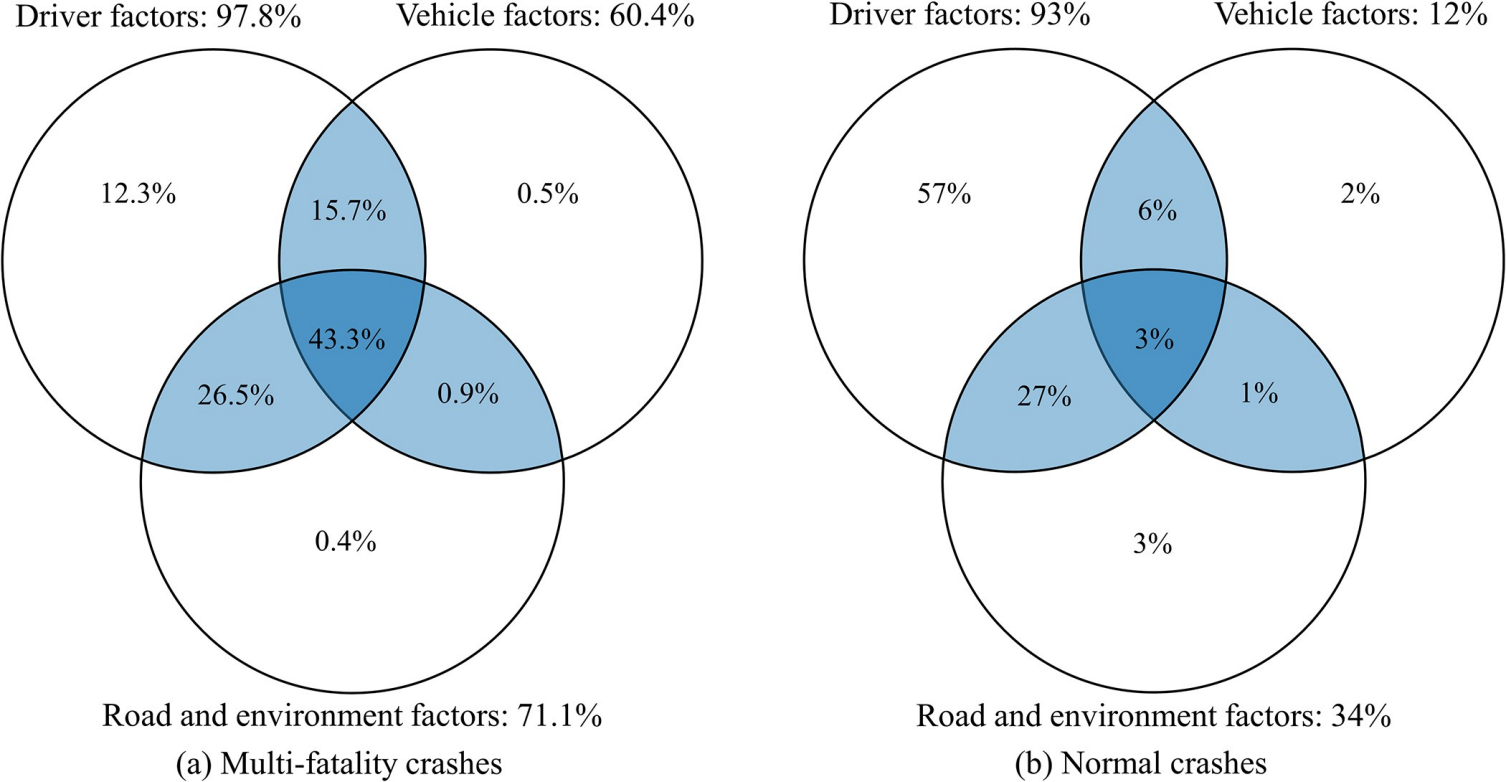
Contributing factors of multi-fatality crashes and normal crashes.

Fig 6(A) shows the complicated relationship of severe fatal crashes in this study to further explore the interactions among variables at the system level of driver-vehicle-road-environment factors. Although the promotion for each factor exceeds 60% in the dataset, only 12.3% of crashes are caused solely by driver factors, and almost no severe crashes are caused solely by road environment or vehicles. A Venn diagram of common accidents proposed by Lum and Reagan is also shown in Fig 6(B) to compare the mechanisms between serious and normal accidents [49]. The proportion of severe accidents caused by human-vehicle-road-environment (43%) is much higher than that of general accidents (3%). This findings firstly demonstrates that the crash mechanisms involving multi-fatality crashes and their interactions are more complex than those for normal crashes from a system-level perspective, which supports the conjecture from previous studies [33].

The results of the association rules are consistent with the differences between severe and normal crashes under different conditions. Regarding road environment, some studies have reported that high-level highways are more prone to fatal crashes owing to their high posted speed limits [50]. However, overturning or falling on low-level highways often causes multiple fatalities due to the lack of roadside protection. The potential causes of this phenomenon may be related to the high proportion of mountainous areas and certain highway construction standards in China. Similarly, in terms of vehicle factors, the proportion of bus-involved crashes caused by passenger overloading in multi-fatality crashes is far higher than that in general accidents. Additionally, there are significant differences between serious fatal and common accidents affected by traffic conditions. Previous studies have found that the proportion of normal accidents under free-flow conditions is much lower than that under unstable flow or congestion conditions [47]. However, RGS and association rules indicate that severe single-vehicle crashes are more likely to occur under free-flow traffic conditions caused by overspeed and driver negligence.

Therefore, several preventive measures can be implemented based on the potential issues presented in this study. Avoidance of inconsistent geometric designs, such as down-curve or sharp curve alignment, and linear guidance facilities may be effective in reducing roadside crashes [46]. Specifically, because of the potential safety improvement, it is recommended to correct superelevation transition sections by the roadside conditions when roadways are repaved, which can minimize the operating speed reduction from tangents to horizontal curves, and avoid low-operating speed curves following long tangents [51]. For accident-prone locations, the installation of integral energy-absorbing guardrails is advisable, especially on low-level highways, to reduce potential vehicle rollover and falling.

In terms of the strong association between vehicle overloading and severe casualties, strict supervision policies must be implemented to regulate the overloading of large buses and heavy trucks. Traffic agencies can strengthen vehicle condition testing and enhance on-board speed monitoring facilities to improve the operational quality of buses considering the potential risk of braking or steering failure [52,53]. In terms of environmental factors, according to a recent study by FHWA, an improvement in surface friction can reduce nearly 70% of accidents caused by slippery roadways [54]. Thus, advanced permeable pavements and skid-resistant pavements are appropriate strategies for implementation in pluvial regions. Also, the severity and frequency of crashes during the nighttime can be mitigated if designers provide enough lighting and induction facilities on segments that have poor visibility at night [55].

Considering that 97.8% of multi-fatality crashes are associated with driver factors, increasing traffic safety propaganda for drivers is prudent to emphasize the adverse effects of improper driving behaviors. The installation of safety devices such as rumble strips and roadway reflectors at the shoulders and the median, could help keep the driver alert [56]. Roadway engineers should identify their crash-hotspots for the installation of the rumble strips and warning signs to remind drivers who may be driving towards such directions. Given that speeding is the most frequent factor involved in crash rules, an automated section speed control system can be established as an effective measure to increase drivers’ compliance with speed limits [57,58].

With regards to the interactions between multiple factors, the utilization of integrated countermeasures in system-level is judicious. With the rapid development of intelligent transportation systems, some active technologies, such as connected and autonomous vehicles (CAV) and advanced driving assistance system (ADAS), have emerged to improve the road capacity and driving safety [59–61]. The previous study assessed the safety benefit of CAV and found 12–47% reduction in traffic conflict at 25% market penetration rate [62]. At the same time, it is suggested to promote ADAS to help drivers deal with unexpected events in unfamiliar conditions [63]. The combination of advanced active safety measures and the proposed crash patterns may reduce the risk of multiple fatalities. It is worth noting that the impact of CAV platoon strategy on driving behavior is still controversial, and the patterns between different variables under mixed traffic still need further investigation.

## 7. Conclusions

Severe roadway multi-fatality crashes are a vital issue for road safety in China. In this study, a case study is conducted using data from 1068 severe fatal crashes in China from 2015 to 2020, and 1452 interesting rules are generated using the Apriori algorithm. Several modular RGSs are constructed to reflect the interactions and patterns between different crash factors. Based on the results and discussion, the following conclusions are drawn:

1. Multi-fatality crashes result from complex interactions among drivers, vehicles, roadways, environment, and crash factors; moreover, improper operation, passenger overloading, fewer lane numbers, mountainous terrain, and ROR crashes are the key variables for each aforementioned factor, respectively.
2. The patterns of different crash characteristics are quite different. The main characteristics of ROR crashes in mountainous areas include inappropriate alignment designs, vehicle defects, and driving violations. The combination of driver errors, overspeed, specific traffic conditions, poor lighting conditions, and slippery surfaces significantly contributes to severe double- and multi-vehicle crashes.
3. The factors involving multi-fatality crashes and their interactions are more complex than those involving normal crashes at the system level. A combination of multiple factors contributes to the excessive frequency and severity of multiple crashes. Therefore, the utilization of integrated countermeasures is judicious.

The contributions of this paper are reflected in three aspects. First, using ARM, many interesting rules are obtained to investigate the contributory factors of serious roadway fatalities and the hidden interactions among them. Second, with rule visualization analysis, targeted safety countermeasures are proposed from the perspective of road design, engineering measures, safety technology, and supervision strategy. Finally, this study explores the applicability of graph structures to the interpretation of association rules and verifies the feasibility of modular networks in pattern division, providing interpretable information for traffic safety analysis.

Notably, the relatively high thresholds for lift and confidence lead to few interesting rules with plain terrain as consequent. As a potential issue for further study, we aim to conduct a causation analysis for plain roadway crashes. Furthermore, considering the gradual deployment of ITS in the near term, the patterns of mixed CAV traffic conditions related to traffic safety and their intrinsic associations need to be deeply explored. In addition, network-based visualization of association rules can be further explored using meaningful graph attributes and node properties. Therefore, further insight is required to form a more comprehensive graph structure approach for association interpretation. Based on graph theory, RGS can provide an understandable perspective for traffic analysis.

## Supporting information

S1 Table Raw data of 1068 fatal crashes. (CSV)

S2 Table 1452 association rules of fatal crashes. (CSV)
